# Impact of compensatory growth on survival in newborn kittens

**DOI:** 10.3389/fvets.2024.1419383

**Published:** 2024-07-03

**Authors:** Amélie Mugnier, Virginie Gaillard, Sylvie Chastant

**Affiliations:** ^1^NeoCare, ENVT, Université de Toulouse, Toulouse, France; ^2^Royal Canin Research Center, Aimargues, France; ^3^Ecole Nationale Vétérinaire d’Alfort, BREED, Maisons-Alfort, France

**Keywords:** early growth, kitten, low birth weight, management, threshold, neonatal mortality, CART analysis, maternal programming

## Abstract

In the feline species, the mortality of live-born kittens over the first 2 months of life is around 10%. Although some losses are inevitable, progress in veterinary medicine and improved knowledge of feline neonatalogy should make it possible to reduce them to a lower level. The objectives of this study were: (i) to describe early growth in kittens, and (ii) to assess whether low birth weight kittens develop compensatory growth during the first week of life and if so, whether compensatory growth is associated with increased chances of survival. Using data collected from 5,504 kittens born in 193 different French catteries, five growth rates were calculated to reflect the growth of kittens during the first week after birth. Low birth weight kittens had higher growth rates than normal birth weight kittens. In addition, low birth weight kittens whose early growth was in the lowest 25% had a significantly higher 0–2 months mortality rate than all the other groups. Weight loss (or lack of weight gain) between birth and Day 2 was identified as a risk factor for 0–2 months mortality whatever the birth weight category. Finally, critical early growth thresholds were determined separately for low and normal birth weight kittens. These figures could help caregivers to validate the adequacy or inadequacy of kitten early growth. They will be able to quickly identify and provide appropriate care for the kittens whose growth is deemed insufficient in order to improve their chances of survival.

## 1 Introduction

In many mammalian species, including cats, ranges of birth weight are wide (1) and low birth weight (LBW) has been identified as a major risk factor for neonatal mortality (2–4). It has been shown that LBW newborns are less toned at birth, with lower energy reserves, putting them at greater risk of hypoglycemia and hypothermia than their siblings and making them particularly dependent on colostrum intake (5–7).

In general, LBW newborns suffer from intra uterine growth retardation (8, 9) resulting from an adverse maternal, placental, fetal, genetic environment (10). Due to energy restriction during fetal development, Hales and Barker suggested that human fetuses develop early-life metabolic adaptations to promote survival, leading to a so-called “thrifty phenotype” (11). This high fetal nutritional efficiency, once placed in a post-natal high availability of nutrients would promote increased postnatal growth, also called compensatory growth (12). This phenomenon has been widely described in various species such as rodents, humans, cattle and pigs (12–14) although it is not systematic. In some studies, LBW piglets have been described as remaining smaller than their littermates throughout the production cycle (15, 16).

In the context of maternal programming, the aim of this study was to assess the relationships between birth weight, early growth and survival over the first 2 months of life in the domestic cat. First, using a large dataset of kitten weights, we wanted to compare early postnatal growth in LBW and normal birth weight (NBW) kittens. Secondly, we explored the link between birth weight category, early growth pattern and chances of survival of the kittens. As the majority of kitten deaths occur in the first week of life (17, 18), this work focused on growth between birth (Day 0) and Day 7, subsequently named early growth.

## 2 Materials and methods

### 2.1 Study population and data

The current study used the same data collected as the one described in Mugnier et al. (1). These data were recorded by breeders and retrospectively collected for the study between 2016 and 2020 on a voluntary basis. Recorded data used for the present work included breed (only purebred kittens were considered), weights of kittens measured throughout the first week after birth (from Day 0 to Day 7), survival status at 2 months of life (alive or dead) and, day or period of death when available. Only kittens born alive with birth weight provided, with known status at 2 months of age and born since year 2000 were considered.

Kittens of the final dataset were classified as low or normal birth weight (LBW or NBW) using breed-specific thresholds defined previously (4). In the present study, all kittens whose birth weight was below the Threshold 1 were classified as LBW, this category thus grouping LBW and very low birth weight (VLBW) defined previously. From weights measured over the first week of life, five growth rates (GR) were calculated: D0–D1 (GR 0–1), D1–D2 (GR 1–2), D0–D2 (GR 0–2), D2–D7 (GR 2–7), and D0–D7 (GR 0–7). Growth rate between Day x and Day y (GR x-y) was calculated as [(weight at Day y – weight at Day x) ÷ weight at Day x] x 100. Growth rates from birth (i.e., GR 0–1, GR 0–2, and GR 0–7) were classified into quartiles: group 1: ≤ first quartile value (Q1), group 2: between Q1 and Q2, group 3: between Q2 and Q3, and group 4: > Q3.

**Figure 1:**
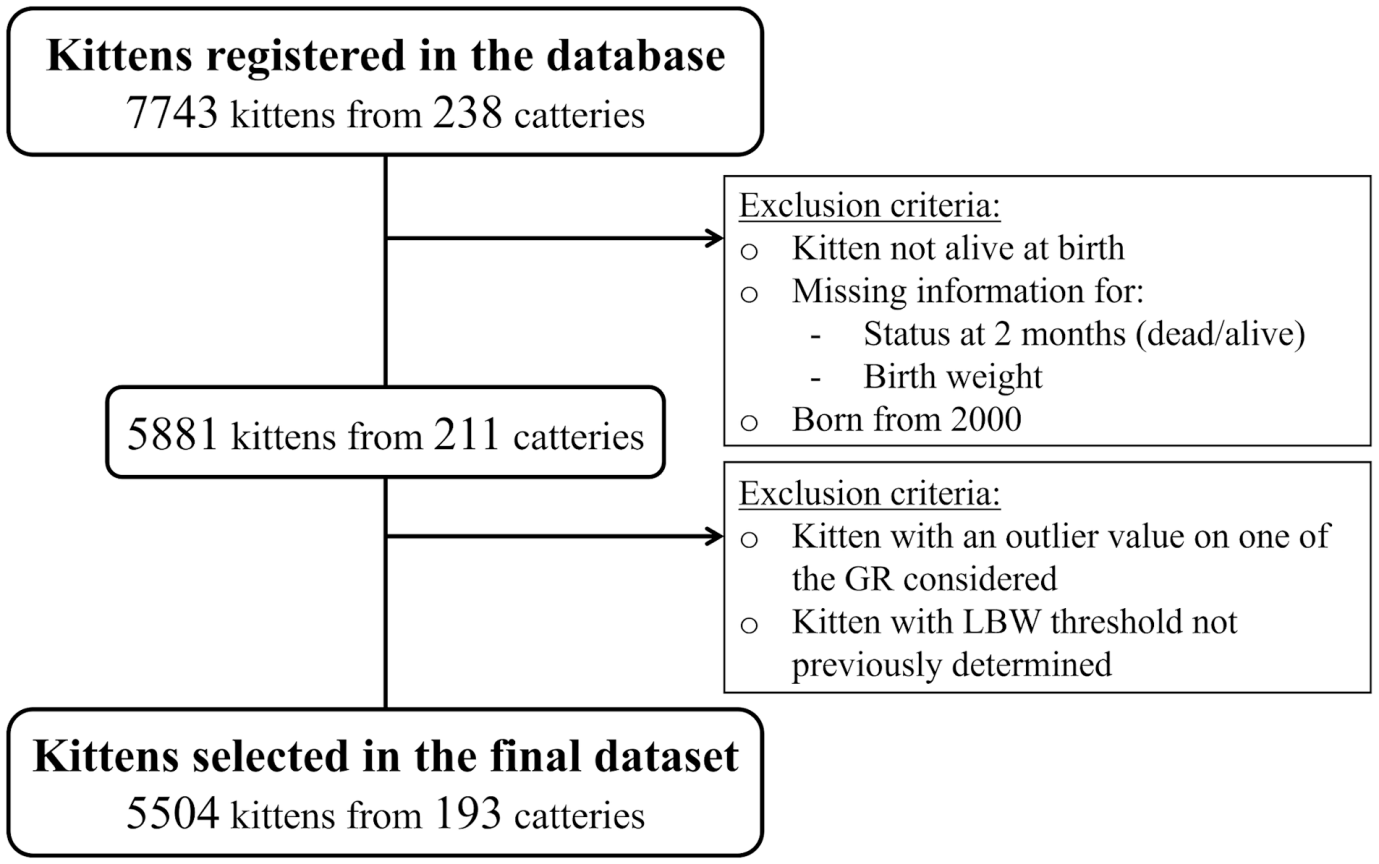
Data selection process.

Prior to analysis, a second round of data selection was implemented to remove GR outliers (i.e., values considered abnormal in relation to the expected distribution) that can significantly impact the statistical analysis (19). GR values were considered outliers if they were higher than the 75th centile plus 1.5 times the interquartile range (IQR), or lower than the 25th centile minus 1.5 times the IQR (Tukey’s method). Kittens with an outlier on one of the GR studied were excluded. A missing value on one of the GR studied was not considered to be an exclusion criterion. Thus, kittens with a missing value were kept for the analysis of the other GR. The entire data selection process is described in Figure 1.

### 2.2 Early growth analysis

After a global description, the five growth rates were compared between LBW and NBW kittens using a Welch *t*-test.

The second part of the analysis aimed to assess the cumulative impact of birth weight and early growth on 0–2 months mortality. The analysis was conducted on three distinct periods: impact of birth weight and GR 0–1 on mortality between Day 1 and 2 months of age; impact of birth weight and GR 0–2 on mortality between Day 2 and 2 months of age; impact of birth weight and GR 0–7 on mortality between Day 7 and 2 months of age. For each assessment, we built a Generalized Linear Model (GLM, function glm from the R package stats) using a logit link function. The outcome was the mortality over the considered period (binary variable) with the two explanatory variables “Birth weight” (LBW or NBW) and “Growth rate” (four quartiles) as well as the interaction between them. *Post hoc* comparisons were made using the R package emmeans (20) to obtain Estimated Marginal Means (EMMs).

The last part of early growth analysis was aimed at defining GR thresholds used to identify kittens characterized by an increased risk of death throughout the first 2 months after birth.

Classification trees (CART) were used to identify kittens at increased risk of mortality during their first 2 months after birth. It is a nonlinear and nonparametric model based on the recursive partitioning method which consists in repeatedly partitioning the data into several subgroups, so that the results in each final subgroup are as homogeneous as possible (21, 22). The method provides the rule (here, cut-off value) used for predicting the outcome variable (here, status dead or alive at 2 months). In this study, CART analyses were used to determine thresholds for the five GR calculated (GR0–1, GR1–2, GR 0–2, GR 2–7, and GR 0–7). NBW and LBW kittens were analyzed separately, leading to the potential identification of 10 threshold values. In this study, the dataset was class-imbalanced, as the number of alive kittens at 2 months is much higher than those dying between the considered period. To avoid a bias in the classification results, a cost-sensitive CART approach was used by defining the cost of a false negative (a kitten dying before 2 months of age but classified as a survivor by the algorithm) as 10 times higher than the one of a false positive. For each threshold, the Gini index was used as the splitting method and a 10-fold cross-validation was used as the method for testing the trees obtained. Final threshold was defined as the median of the 10 values obtained. Finally, performances of the determined thresholds were calculated with sensitivity (Se), specificity (Sp), positive and negative predictive values (PPV and NPV).

### 2.3 Data management and analysis

All statistical analyses were performed using R software, version 4.2.1 (23). The package ggplot2 (24) was used for data visualization, dplyr (25) and tidyr (26) for data manipulation and finally, rpart (27) and caret (28) to perform CART analyses. A value of *p* < 0.05 was considered statistically significant and statistical uncertainty was assessed by calculating 95% confidence intervals (95% CI).

## 3 Results

### 3.1 Description of the population

Data from a total of 5,504 live-born kittens from 1,499 litters born in 193 French catteries were used for the analyses. Litters were born between 2000 and 2020 with 75% of them born from 2010 onwards. Fifteen breeds were represented in the dataset, including the top-ten breeds owned in France (29). The median number of kittens included per breed was 274, ranging from 108 for the Russian Blue/Nebelung to 880 for the Maine Coon. The global mean litter size (number of born alive kittens) was 4.5 (SD: 1.5) kittens and sex ratio was calculated as 1.1 (2,760 males vs. 2,416 females, sex unknown for 328 kittens). Birth weights ranged from 36 g (a Persian/Exotic kitten) to 182 g (a Norwegian Forest kitten) with a median at 101 g (IQR: 89–115). Among the 5,504 kittens selected, 1,075 (19.5%) were classified as LBW and 4,429 (80.5%) as NBW. Birth weight threshold values, defining LBW by breed, are presented in Supplementary Table S1.

### 3.2 Description of early growth

Growth rates over the first week are described in Table 1. For all the GR, values were spread out around the averages, which were all positive, even though 11.4% (544/4,459), 5.7% (271/4,716) and 0% of the kittens had lost weight between birth and Day 1, birth and Day 2 and birth and Day 7, respectively. Daily GR over the first week after birth was on average 9.5% (SD: 5.9) with statistically significant differences between days (from 7.3% for GR 6–7 to 11.5% for GR 2–3; *p* < 0.001, paired t-test) but with negligeable to small size effect (Cohen’s d, absolute values between 0 and 0.6) (30).

**Table 1:** Description of early growth rates depending on kitten birth weight.

	Mean growth rates, % (± SD)			Comparison^a^	
	All kittens	LBW	NBW	*p*	Effect size
GR 0–1	9.4 (± 8.8) (*n* = 4,756)	9.8 (± 10.2) (*n* = 925)	9.4 (± 8.4) (*n* = 3,831)	ns	–
GR 1–2	9.5 (± 7) (*n* = 4,591)	10.5 (± 8.4) (*n* = 868)	9.3 (± 6.5) (*n* = 3,723)	***	0.16 (negligible)
GR 0–2	19.9 (± 13) (*n* = 4,716)	21.8 (± 15.3) (*n* = 891)	19.5 (± 12.3) (*n* = 3,825)	***	0.17 (negligible)
GR 2–7	57.7 (± 14.4) (*n* = 4,384)	63.0 (± 16.5) (*n* = 804)	56.5 (± 13.7) (*n* = 3,580)	***	0.43 (small)
GR 0–7	89.3 (± 25) (*n* = 4,692)	99.9 (± 30.4) (*n* = 844)	86.9 (± 23) (*n* = 3,848)	***	0.48 (small)

SD, standard deviation; n, number of kittens included in the analysis; GR, growth rate; LBW, low birth weight kittens; NBW, normal birth weight kittens.

GR x-y = growth rate between Day y and Day x, calculated as [(weight at Day y – weight at Day x) ÷ weight at Day x] × 100.

a Welch test result for the comparison between LBW and NBW kittens: *** for *p* < 0.001, ** for *p* < 0.01,* for *p* < 0.05, and ns for non-significant; effect size was evaluated using Cohen’s d.

Growth rates for the five periods were compared between LBW and NBW kittens (Welch t-test). Except during the first day after birth (no difference in GR 0–1), LBW kittens grew significantly faster than NBW during the first week of life (all *p* < 0.001, Table 1) but with negligible to small effect size (Cohen’s d, absolute values between 0.2 and 0.5).

### 3.3 Relationship between birth weight, early growth and kitten mortality

A total of 7.4% (408/5,504; 95% CI [6.7, 8.1]) of live-born kittens died during the first 2 months of life with most deaths (82%, 335/408) occurring during the neonatal period (between birth and Day 21).

**Figure 2:**
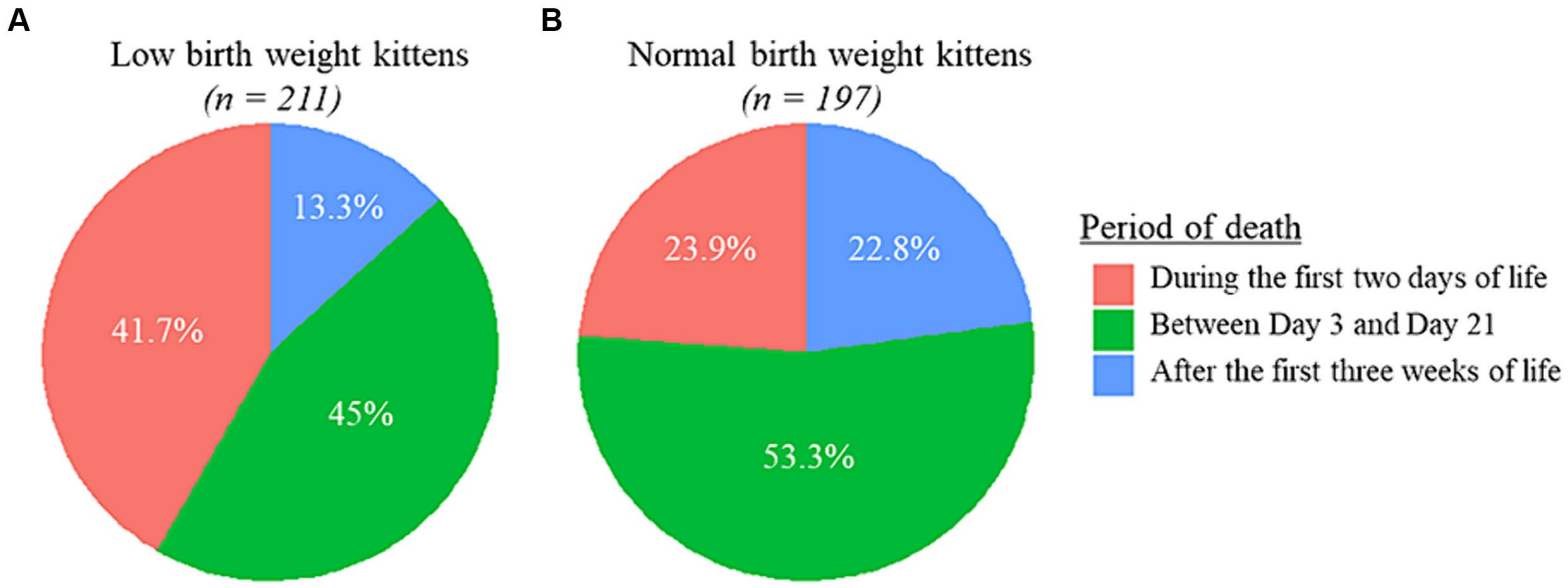
Kitten deaths during the first 2 months of life: distribution by periods according in **(A)** low birth weight kittens (*n* = 211) and **(B)** normal birth weight kittens (*n* = 197).

The overall mortality rate during the first 2 months of life was 4.4 times higher for LBW than for NBW kittens (19.2% (95% CI [17.3, 22.1]) vs. 4.4% (95% CI [3.9, 5.1]); Chi-square test, *p* < 0.001). In addition, the distribution of cases over the period considered was also different depending on birth weight categories (Figure 2): LBW kittens died significantly more frequently during the two intervals covering the neonatal period (0–2 days and 3–21 days) and significantly less after the third week of life than NBW kittens (Chi-square test, all *p* < 0.001).

**Figure 3:**
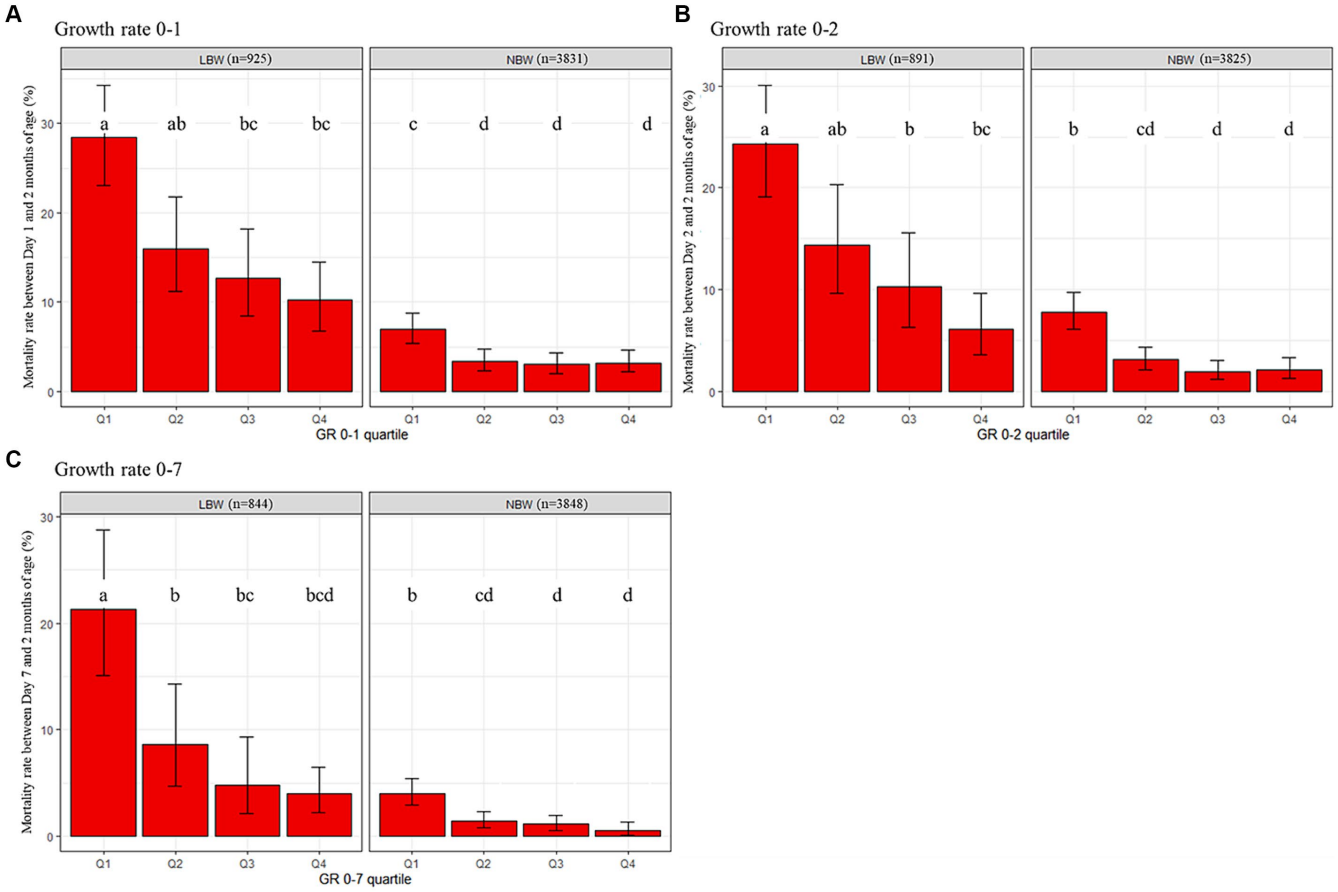
Cumulative impact of birth weight and early growth on mortality rates of kitten during the first 2 months after birth **(A)** from Day 1; **(B)** from Day 2; **(C)** from Day 7. Growth rate categories were constructed based on quartile values calculated at species-level. LBW, low birth weight; NBW, normal birth weight; GR, growth rate. The error bars represent statistical uncertainty (95% binomial confidence intervals). Different letters at the top of the bars indicate significant differences in mortality rates (Generalized Linear Model followed by pairwise *post hoc* comparisons).

Mortality rates of LBW and NBW kittens depending on their early GR category are presented in Figure 3. Three different periods were considered: Day 0 to Day 1, Day 0 to Day 2 and the first week of life (Day 0 – Day 7). The mortality rates of LBW kittens were significantly affected by the GR. LBW with GR in Q1 were the kittens most at-risk, with 1.8-, 2.2-, and 2.5-times higher mortality rates, respectively, for GR 0–1, GR 0–2, and GR 0–7. Growth rates above the median for GR 0–1 and above the first quartile for GR 0–2 and GR 0–7 reduced the mortality rate of LBW kittens to rates observed in Q1 GR NBW kittens. In NBW kittens, GR also affected mortality rate, as Q1 GR NBW kittens died in a higher proportion than in the three other GR groups.

### 3.4 Identification of early growth thresholds

Thresholds were searched in both subpopulations (NBW and LBW) separately using cost-sensitive classification trees (Table 2).

**Table 2:** Thresholds of early growth rates discriminating for mortality rate over the first 2 months of life in kittens (CART analysis).

Birth weight category	Growth rate	*n*	Growth rate threshold, %	Mortality over the first 2 months of age				Characteristics of the threshold			
				For all kittens included	For kittens with growth rate below the threshold	For kittens with growth rate equal or greater than the threshold	Mortality multiplication term between NBW and LBW	Se	Sp	PPV	NPV
LBW	GR 0–1	925	1.3	17.2%	32.5% (*n* = 203)	12.9% (*n* = 722)	2.5	0.42	0.82	0.33	0.87
	GR 1–2	868	8.6	13.9%	23.3% (*n* = 339)	7.9% (*n* = 529)	2.9	0.65	0.65	0.23	0.92
	GR 0–2	891	9.5	13.7%	27.9% (*n* = 197)	9.7% (*n* = 694)	2.9	0.45	0.82	0.28	0.90
	GR 2–7	804	42	8.2%	28.4% (*n* = 81)	5.9% (*n* = 723)	4.8	0.35	0.92	0.28	0.94
	GR 0–7	844	58	8.1%	36.6% (*n =* 71)	5.4% (*n* = 773)	6.8	0.38	0.94	0.37	0.95
NBW	GR 0–1	3,831	1.1	4.1%	8.8% (*n* = 616)	3.2% (*n* = 3,215)	2.8	0.34	0.85	0.09	0.97
	GR 1–2	3,723	1.3	3.7%	13.4% (*n* = 403)	2.5% (*n* = 3,320)	5.4	0.39	0.9	0.13	0.98
	GR 0–2	3,825	1.7	3.7%	13.8% (*n* = 290)	2.9% (*n* = 3,535)	4.6	0.28	0.93	0.14	0.97
	GR 2–7	3,580	56	1.8%	3.1% (*n* = 1723)	0.7% (*n* = 1857)	4.4	0.80	0.52	0.03	0.99
	GR 0–7	3,848	67	1.8%	5.3% (*n* = 718)	1% (*n* = 3,130)	5.3	0.54	0.82	0.05	0.99

Thresholds were determined separately for LBW and NBW kittens.

LBW, low birth weight; NBW, normal birth weight; n, number of kittens included; GR, growth rate; ND, non determined; Se, sensitivity; Sp, specificity; PPV, positive predictive value; NPV, negative predictive value.

GR x-y = growth rate between Day y and Day x, calculated as [(weight at Day y – weight at Day x) ÷ weight at Day x] × 100.

Depending on the GR and the birthweight category evaluated, mortality rates between kittens above and below the defined threshold were between 2.5 and 6.8 times higher in those with the lowest GR. Differences in mortality between the two groups (below or above the threshold) increased with the age of the kittens used for the analysis (2.5 vs. 4.5 higher for GR 0–1 vs. GR 2–7 respectively). Considering reduction in points of percentage, the impact of low growth (GR below the thresholds) on mortality rate was dramatically (4–6 times) higher in LBW than in NBW at all periods of time (Table 2; Figure 3). In both subpopulations, the impact of low growth increased with age.

## 4 Discussion

As an altricial species, neonatal period of cats is challenging. Kittens must transit from intra to extra-uterine life, affecting several physiological functions. Nutrition and metabolism are drastically modified, switching from a passive placental to an active digestive nutrition. Considering the high postnatal mortality rates described in kittens (17, 18, 30, 31), improving knowledge on the neonatal period, management practices and survival determinants is mandatory. This study was conducted on a large population of kittens (*n* = 5,504) from various breeds (*n* = 15) in which mortality rate from birth to 2 months of age (7.4%; 95% CI [6.7, 8.1]) was in the range of previous works [e.g., 7.9% (31) and 9.1% (17)].

As the impact of early growth on mortality rates over the two first months of life is well described, the first objective of this study was to deeply explore this outcome in the domestic cat. In this work, we examined five unique growth rates (GR) to holistically characterize the initial week of life, initially as an entire period (GR 0–7). Subsequently, we performed distinct analyses for the colostral phase (GR 0–2 and GR 2–7). Moreover, we concentrated specifically on the early neonatal period (GR 0–1 and GR 1–2), considered particularly crucial for kitten survival. In our study average GR, whatever the period considered from birth to Day 7, were positive (Table 1) suggesting the absence of early weight loss, as described in humans (32, 33). It is interesting to note that, as previously described in humans (34), we observed significant standard deviations around the mean, underlining the great variability in growth between kittens from birth.

In 1934, Hall and Pierce reported significantly higher GR for LBW kittens compared with NBW during the first week of life (35). However, this observed difference was not further investigated. In our study, we found a similar pattern, with a 13-percentage point difference in GR between LBW and NBW kittens during the first week (GR 0–7). This finding suggests the possibility of a compensatory growth in LBW newborns, potentially as a response to intra-uterine growth restriction. However, this interpretation warrants caution. In our sample, it is plausible that LBW kittens received additional care, such as enhanced suckling stimulation and supplemental feeding with milk formulas. Such interventions could contribute to the observed compensatory growth. Therefore, the observed growth acceleration in LBW kittens could be attributed not only to their birth weight status but also to the extra care they received, given their perceived vulnerability by breeders. Indeed, LBW lambs, piglets and puppies are often less able to develop skills allowing them to survive such as efficient sucking behavior (36–39), and this has also been described in kittens (40). Further research is needed to disentangle these potential influences on early growth patterns in kittens and suspected compensatory growth in LBW. However, investigating spontaneous growth would require stopping any assistance or any additional feeding, leading to ethical issues.

The amplitude of the compensatory growth observed for LBW kittens over their first week of life is also associated with a reduction of 0–2 months mortality rates (Figure 3). The mortality rates of LBW kittens decrease with increased GR, whatever the period. Whereas LBW kittens with the lowest GR (Q1 and Q2) have systematically higher mortality rates, kittens with the highest GR (Q3 and Q4) reach mortality rates equivalent to those of NBW kittens with the lowest GR (Q1). Nevertheless, even when benefiting from the highest GR, LBW newborns die in higher proportions than most NBW (Q2, Q3, and Q4). This suggests that the early promotion of GR can help reducing losses among LBW kittens but cannot reverse the deleterious impact of reduced intra-uterine growth. Research in piglets suggests that impaired intestinal development in LBW could lead to reduced nutrient utilization (41). Further works are warranted to compare organ development, digestion capacity and nutrient absorption between LBW and NBW kittens. Finally, our work supports that NBW kittens should also be monitored for weight gain over the first week of life, as suggested by the increased mortality in those with GR in the lowest 25%.

Growth monitoring has been proposed as one of the tools for ensuring neonatal follow-up in many species [canine (42), human (34, 43), ovine (44, 45) and even non-domestic species (46, 47)]. The importance of daily weighing from birth is clearly confirmed by the results of the present study. This easy-to-implement criterion allows the early detection of difficulties to adapt to extra-uterine life and/or underlying pathologies (48). In feline breeding, neonatal weighing is a common practice, with about three quarters of the breeders performing close monitoring of kitten weight over the three first weeks of life (49). Despite this close follow-up, catteries still face a high mortality rate during the first weeks of life (around 10% of live-born kittens die over the two first months) (17, 18, 31, 50). One hypothesis could be the absence of guidelines or thresholds to assess whether growth is normal or not. To provide a decision-making tool, the last part of the current study was aimed at defining thresholds to assess the adequacy of kitten early growth. In this work, thresholds were defined for the domestic cat as a whole, as the first operational step, with possible later refinement by taking into account breed or sex, as suggested in dogs (51, 52). GR thresholds were defined regarding the mortality risk over the two first months of life and by taking into account intra-uterine growth based on birth weight category (LBW or NBW).

For growth between birth and Day 1 (GR 0–1) the thresholds identified for both LBW and NBW were close to 1% (Table 2). For GR 1–2 and GR 0–2 and important difference between the thresholds defined for LBW and NBW was observed (threshold values close to 1% for NBW vs. higher than 8% for LBW). All together, these results suggest that, as in dog, another polytoccous altricial species, newborns should at least not lose weight from birth to Day 2 to maximize their chances of survival, whatever their birth weight. These first 2 days of life include the colostrum intake (during the first 12 to 16 h of life) which is essential to the health of newborns as it provides immunoglobulins, energy and nutrients (53, 54). In addition, the highest thresholds defined for LBW for GR 1–2 and GR 0–2 suggest the need of compensatory growth (to balance their reduced intra uterine growth) to increase their chances of survival right from the very first days of life (Table 2).

As expected, LBW kittens with GR in the lowest 25% (Q1) were the most at-risk category whatever the period of growth considered (Day 0 to Day 1, Day 0 to Day 2 or Day 0 to Day 7). This suggests an additive effect of both intra and early extra-uterine growth on 0–2 month mortality (Figure 3). Thus, care-givers can contribute to increasing survival of LBW kittens by ensuring adequate early growth. Various strategies have been reviewed by De Vos et al. (38) in piglets, a species characterized by a high proportion of LBW newborns. One strategy is to give LBW kittens more time to obtain colostrum, and then milk, without the presence of more competitive littermates or by helping them to cling to the nipples (controlled suckling). Another strategy is to avoid large litters and/or large intra litter variations in birth weight through the adoption of one or more kittens by another queen with a smaller litter (cross-fostering). However, this method can be difficult to implement in the feline species because in general catteries host a limited number of breeding females (55), with unsynchronized queenings, contrary to pig breeding conducted in large groups of females delivering on the same day. Finally, additional milk supply could be proposed to LBW kittens through bottle- or tube-feeding (56, 57). Whatever the strategy chosen, it should be implemented as soon possible as considerable losses occur over the first 2 days of life in LBW kittens (Figure 2).

Compensatory growth appears to be an efficient tool to control 0–2 months kitten mortality. Besides its short term (2 first months of life) beneficial impact, i.e., increased survival, in humans compensatory growth has been suspected of inducing harmful effects on long-term health, with an increased risk of obesity (58), diabetes mellitus and cardiovascular disease (59), and ultimately a reduction in life expectancy (13, 60). This phenomenon might be attributed to the nature of compensatory growth, which appears to favor adipose tissue over muscular or visceral tissue, resulting in altered body composition. During slaughter, the carcasses of LBW piglets exhibited higher fat content and relatively lower muscle proportions compared to their counterparts of similar weight at slaughter, despite being heavier at birth (61, 62). Further research is necessary to investigate changes in body weight and composition among kittens based on their birth weight categories. The first 2 months are a period of critical importance for programming health at adulthood in cats. It has been proposed that this period would be similar to the a “first thousand days of life” in humans (63). The adequacy of growth should be explored not only through its impact on kitten mortality but also on its impact on global adult health (e.g., body condition and metabolic diseases) (64).

## 5 Conclusion

The exploration of factors susceptible to help reduce neonatal mortality in the feline species is warranted. Adequate early growth, which reflects good adaptation to extrauterine life, could help to increase chances of survival, especially for LBW kittens which are considered more at-risk. This study highlighted that kittens with a slower growth rate have higher mortality risk, and even more so in LBW. A compensatory growth was observed in LBW kittens right from the very first days of life, even if its origin remains to be explored as it could be linked to the support of the caregiver. In the last part, we defined GR thresholds that can help veterinarians and breeders to identify impaired early growth, even as we strive to enhance prediction quality through additional data collection. From a practical point of view, growth monitoring will be easier for breeders and veterinarians thanks to the integration of defined thresholds in digital systems providing automatic warnings. Beyond the neonatal period, for surviving kittens, further research is required to study the mid-term and long-term consequences of early growth at adulthood and elderly ages.

## Data availability statement

The raw data supporting the conclusions of this article will be made available by the authors, without undue reservation.

## Ethics statement

The animal studies were approved by Royal Canin Ethical Review Committee, France. The studies were conducted in accordance with the local legislation and institutional requirements. Written informed consent was obtained from the owners for the participation of their animals in this study.

## Author contributions

AM: Conceptualization, Data curation, Formal analysis, Investigation, Methodology, Project administration, Visualization, Writing – original draft, Writing – review & editing. VG: Funding acquisition, Resources, Writing – review & editing. SC: Conceptualization, Investigation, Methodology, Resources, Supervision, Writing – review & editing.
