# Supplementary material for: Impact of compensatory growth on survival in newborn kittens
Source: Front Vet Sci. 2024 Jul 3;11:1419383. doi: 10.3389/fvets.2024.1419383 (PMC11252836; doi:10.3389/fvets.2024.1419383)
Supplement: Supplementary file 1 [file Table_1.DOCX]

Supplementary Material

**Table S1. Birth weight thresholds for discriminating low and normal birth weight kittens by breed.**

| Group | BW threshold (in grams) |
| --- | --- |
| Abyssinian/Somali | 94 |
| Balinese/Mandarin/Oriental/Siamese | 82 |
| Bengal | 84 |
| Birman | 74 |
| British | 87 |
| Chartreux | 100 |
| Egyptian Mau | 104 |
| Maine Coon | 81 |
| Norwegian Forest | 94 |
| Persian/Exotic | 82 |
| Ragdoll | 84 |
| Russian Blue/Nebelung | 86 |
| Scottish/Highland | 77 |
| Siberian | 90 |
| Sphynx | 76 |
